# CVID-Associated Tumors: Czech Nationwide Study Focused on Epidemiology, Immunology, and Genetic Background in a Cohort of Patients With CVID

**DOI:** 10.3389/fimmu.2018.03135

**Published:** 2019-01-22

**Authors:** Pavlina Kralickova, Tomas Milota, Jiri Litzman, Ivana Malkusova, Dalibor Jilek, Jitka Petanova, Jana Vydlakova, Alena Zimulova, Eva Fronkova, Michael Svaton, Veronika Kanderova, Marketa Bloomfield, Zuzana Parackova, Adam Klocperk, Jiri Haviger, Tomas Kalina, Anna Sediva

**Affiliations:** ^1^Department of Allergology and Clinical Immunology, Faculty of Medicine, Charles University and University Hospital in Hradec Kralove, Hradec Kralove, Czechia; ^2^Department of Immunology, Second Faculty of Medicine, Charles University and Motol University Hospital, Prague, Czechia; ^3^Department of Allergology nad Clinical Immunology, Faculty of Medicine, Masaryk University and St Anne's University Hospital in Brno, Brno, Czechia; ^4^Department of Allergology and Clinical Immunology, Faculty of Medicine in Pilsen, Charles University and University Hospital Pilsen, Pilsen, Czechia; ^5^Department of Allergology and Clinical Immunology, Institute of Health, Usti nad Labem, Czechia; ^6^Institute of Immunology and Microbiology, First Faculty of Medicine, Charles University and General University Hospital in Prague, Prague, Czechia; ^7^Department of Clinical Immunology and Allergology, Institute for Clinical and Experimental Medicine, Prague, Czechia; ^8^Department of Pneumology, Regional Thomas Bata Hospital, Zlin, Czechia; ^9^Childhood Leukemia Investigation Prague, Second Faculty of Medicine, Charles University, Prague, Czechia; ^10^Department of Informatics and Quantitative Methods, Faculty of Informatics and Management, University of Hradec Kralove, Hradec Kralove, Czechia

**Keywords:** common variable immunodeficiency, malignancy, lymphoma, gastric cancer, whole exome sequencing

## Abstract

**Background:** Common variable immunodeficiency disorder (CVID) is one of the most frequent inborn errors of immunity, increased occurrence of malignancies, particularly lymphomas, and gastric cancers, has long been noted among CVID patients. Multifactorial etiology, including immune dysregulation, infections, chronic inflammation, or genetic background, is suggested to contribute to tumor development. Here, we present the results of the first Czech nationwide study focused on epidemiology, immunology and genetic background in a cohort of CVID patients who also developed tumors

**Methods:** The cohort consisted of 295 CVID patients followed for 3,070 patient/years. Standardized incidence ratio (SIR) was calculated to determine the risk of cancer, and Risk ratio (RR) was established to evaluate the significance of comorbidities. Moreover, immunophenotyping, including immunoglobulin levels and lymphocyte populations, was assessed. Finally, Whole exome sequencing (WES) was performed in all patients with lymphoma to investigate the genetic background.

**Results:** Twenty-five malignancies were diagnosed in 22 patients in a cohort of 295 CVID patients. SIR was more than 6 times greater in comparison to the general population. The most common neoplasias were gastric cancers and lymphomas. History of Immune thrombocytopenic purpura (ITP) was established as a potential risk factor, with over 3 times higher risk of cancer development. The B cell count at diagnosis of lymphoma was reduced in the lymphoma group; moreover, post-treatment B and T cell lymphopenia, associated with poorer outcome, was found in a majority of the patients. Intriguingly, no NK cell depression was observed after the chemotherapy. WES revealed heterogeneous genetic background among CVID patients with tumors, identifying gene variants associated with primary immunodeficiencies (such as CTLA4, PIK3CD, PMS2) and/or increased cancer susceptibility (including BRCA1, RABEP1, EP300, KDM5A).

**Conclusions:** The incidence of malignancy in our CVID cohort was found to be more than 6 times greater compared to the general population. Gastric cancers and lymphomas were the most frequently diagnosed tumors. ITP was identified as a risk factor for malignancy in CVID patients. WES analysis confirmed a wide genetic heterogeneity among CVID patients. The identified causative or modifying gene variants pointed to errors in mechanisms contributing to both immunodeficiency and malignancy.

## Introduction

Immune control of tumor development and growth requires a functional immune system capable of complex immune responses necessary for recognition and elimination of malignant cells. As such, inborn errors resulting in immunodeficiencies may convey an increased risk of cancer. In general, the spectrum of malignancies in primary immunodeficiency (PID) patients is clearly biased when compared to malignant diseases in the general population. Malignancies in PIDs tend to be restricted to certain oncological entities, and their pathophysiology is often linked to the mechanism underlying the particular immunodeficiency (1). For example, immunodeficiencies associated with gain-of-function mutations in *PIK3* are associated with a high risk of lymphoma (2). The role of this signaling pathway in cancer genesis and immunodeficiency is validated by therapeutic success of targeted PI3K/mTOR pathway inhibition used in activating *PIK3* syndrome, as well as in malignant diseases (3). Similarly, immunodeficiencies arising from developmental defects of stem cells, myeloid cells or lymphocytes are associated with an increased incidence of leukemia or lymphomas, implying errors in the corresponding pathways (1). Recent advances in understanding of molecular mechanisms underlying primary immunodeficiencies, as well as tumors, has provided evidence for such associations. However, in immunodeficiencies that are not yet precisely defined by their molecular/genetic cause, the situation is more complex. Common variable immunodeficiency disorder (CVID) is one of the most frequent forms of antibody deficiencies; yet, its pathophysiology remains largely unknown. The hallmark of CVID is the impairment of the B cell compartment, typically manifesting as a reduction of mature forms of B cells and expansion of less differentiated stages of B lymphocytes (4). The T cell compartment is also usually skewed in CVID, specifically toward terminally differentiated forms, including senescent cells, typically affecting both CD4and CD8 T cells, which are crucial for anti-tumor immunity (5). The mechanisms of B cell involvement in anti-tumor immunity are largely unknown. B cells may promote both pro-tumourigenic responses (e.g., specific subsets of B cells may produce IL-10 or TGF-beta with immunosuppressive properties, B cells may promote tumor genesis and tumor progression by alteration of the angiogenic and proinflammatory microenvironment), as well as anti-tumourigenic responses (e.g., B cells may enhance cytotoxic T cell activity, indirectly mediate antibody dependent cytotoxic mechanisms or serve as professional antigen-presenting cells, initiating the T cell response) (6). Inborn impairment of the B cell lineage, along with T cell dysregulation, may facilitate the genesis of tumors in CVID patients. Furthermore, the immunologic defect is accentuated by recurrent and chronic infections. Chronic viral infections, particularly EBV, are strongly associated with lymphoproliferative diseases and lymphoma (7). Additionally, chronic inflammatory response, *per se*, represents a risk factor for tumor development, especially in patients genetically predisposed to malignancy.

Efforts made to establish the genetic etiology of CVID have thus far been successful in 2–10% of CVID patients (known CVID-associated gene variants are shown in Table 1) (8). Some of the CVID-associated genes represent a clear predisposition to cancer, as described in previously published CVID cohorts; most prominently, these mutations are in genes causing alterations in the NFkB or PI3k pathways or in genes affecting B cell receptors (3, 9, 10).

**Table 1:** List of genes associated with Common variable immunodeficiency (monogenic causes and modifier genes) and their prevalence, adjusted according to Bogaert et al. (8).

**Gene**	**Prevalence (%)**
**MONOGENIC CAUSE OF CVID: 2–10%**	
*PIK3CD*	26.74
*LRBA*	26.74
*CTLA4*	6.42
*NFKB2*	5.35
*TNFRSF7 (CD27)*	4.81
*PIK3R1*	4.81
*ICOS*	3.74
*CD19*	3.74
*IL-21R*	3.21
*IKZF1 (IKAROS)*	3.21
*PRKCD*	2.14
*PLCG2*	2.14
*NFKB1*	1.6
*CR2 (CD21)*	1.7
**MODIFIER GENES: UNKNOWN PREVALENCE**	
*TNFRSF13B (TACI), TNFRSF13C (BAFF-R), MSH2, MSH5, CLECG1, MLH1, RAD50, ORC4L, FCGR2A*.	

Overall, the factors contributing to increased incidence of malignancy in CVID are complex, encompassing genetics, immune response dysregulation, infections, inflammation, and perhaps other not yet elucidated mechanisms.

Here, we present the results of a complex study on a Czech national cohort of CVID patients who also presented with malignancy. National epidemiological data were collected, immune profiles were analyzed and, in a subgroup of CVID patients with lymphoma, Whole exome sequencing (WES) was performed.

## Methods

This study was approved by local ethics committee of Motol University Hospital. Written informed consents were obtained from all enrolled patients.

### Data Collection

Retrospective clinical and laboratory data of 295 enrolled patients were obtained from medical records of national referral centers for the treatment of adult patients with primary immunodeficiency diseases. The collected data covered the period from 1997 to 2016. They included a total amount and a length of surveillance of all CVID patients fulfilling ESID/PAGID criteria, a number of CVID cancer patients and patient-specific data: year of birth, age at first symptoms associated with CVID and their nature, clinical comorbidities, age at cancer diagnosis, type of cancer, therapeutic regimens, survival rates, cause of death (if applicable), and specific details of cancer diagnostics.

A more detailed set of clinical data was obtained from 11 CVID patients with cancer and from 160 randomly selected cancer-free CVID patients, who represented the reference group. This cohort included 95 females and 65 males with a median age of 48 years (range 19–88). Czech general population data on occurrence of malignancy were obtained from the Czech National Cancer Registry and covered the period from 1994 to 2014 (the last available reports).

### Epidemiology

Prevalence and SIR were calculated to express the probability of cancer diagnosis in a CVID cohort compared to the general population. RR was used to assess the significance of comorbidities in CVID patients with cancer. Confidence intervals (95% CIs) were determined for both parameters. All results for which number 1 was beyond the 95% CI were accepted as statistically significant (11).

### Immunophenotyping

Serum levels of IgM, IgG, IgA were evaluated by nephelometric method using Image 800 systems (Beckman-Coulter, Brea, CA, USA). Basic lymphocyte subpopulations, including T cells, T helper cells, T cytotoxic cells, B cells and NK cells, were distinguished by FACS based on the expression of specific cell surface membrane markers using fluorochrome-conjugated monoclonal antibodies CD3-FITC, CD4-PE/Cy, CD8-APC/Cy,CD19-APC, CD16-PE, CD56-PE; KOMBITEST, Exbio, Prague, Czech Republic. B cell subpopulations (including CD21low, naive, transitional, marginal zone-like, class-switched cells, and plasmablasts) and T cell subpopulations (recent thymic emigrants-RTE, naïve, central memory-CM, effector memory-EM, effector memory expressing CD45RA-TEMRA, activated T cells) were performed using antibody-fluorochrome conjugates: CD45-APC-H7, CD3-APC, CD4-PerCP-Cy5.5, CD16-PE, CD56-PE, TCRgd-PE-Cy7, CD38-FITC, CD21-APC, IgM-FITC, CD8-Horizon V-500, CD45RA-PE-Cy7 (BD Biosciences, San Jose, CA, USA), CD5-PE,CD8-FITC, CD27-Brilliant Violet 421, IgM-Brilliant Violet 510, IgD-PerCP-Cy5.5, CD4-Brilliant Violet 510, CD62L-Brilliant Violet 421, HLA-DR-PerCP-Cy5.5 (Biolegend, San Diego, CA), CD19-PE-Cy7, CD24-PE, CD24-APC-Alexa Fluor 750, CD8-APC-Alexa Fluor 750 (Beckman Coulter, Miami, FL, USA), CD27-Pacific Blue, CD38-Alexa Fluor 700, CD45RO-FITC, CD31-PE, CD4-Alexa Fluor 700 (Exbio, Vestec, Czech Republic), CCR7-PE (MiltenyiBiotec, BergischGladbach, Germany), CD3-PerCP-Cy5.5 (Affymetrix eBioscience, ThermoFisher Scientific, Waltham, MA, USA), and IgD biotin (SouthernBiotech, Birmingham, AL, USA) followed by Streptavidin-Qdot 605 (Invitrogen, ThermoFisher Scientific, Waltham, MA, USA).

The absolute and relative counts were assessed for all subpopulations. Examinations of the basic subpopulations and immunoglobulin levels were performed at the time of diagnosis of CVID and at the time of diagnosis of lymphoma. The B and T cell subpopulations were measured prior to the genetic testing. All parameters were compared to the control cohort of 20 randomly selected CVID patients without lymphoma. All obtained data were statistically evaluated. A non-parametric Mann-Whitney test was used to compare independent samples, and a non-parametric Wilcoxon matched-pairs signed rank test was used to compare dependent samples; differences of *p* < 0.05 were regarded as significant. Median and 95% CIs were calculated for all analyzed parameters.

### CTLA-4 Expression

CTLA-4 expression was assessed in patient with novel mutations using FACS. Intracellular CTLA-4 was detected 16 h following anti-CD3/CD28 stimulation (Dynabeads, Thermo Fisher Scientific, MA, USA) using CTLA4-APC antibody together with the FOXP3 Fix/Perm Buffer set (BioLegend, San Diego, CA, USA) in CD4+CD127dimCD25+ T regulatory cells (Tregs). CD45-APC-H7, CD4-Brilliant Violet 510, and CD127-Brilliant Violet 421 (BD Biosciences, San Jose, CA, USA), CD25-PE-Cy7 and CD8-FITC (Exbio, Vestec, Czech Republic) antibodies were used for detection of Tregs.

### Whole Exome Sequencing

Sequencing libraries were prepared using a SureSelectXT Human All Exon V6+UTR kit (Agilent Technologies, Santa Clara, CA) from DNA isolated from patients' peripheral blood with a QIAamp DNA Blood Mini Kit (Qiagen, Hilden, Germany). Sequencing was performed by our facility on the NextSeq 500 (Illumina, San Diego, CA) instrument according to the manufacturer's protocols. The reads in resulting Fastq files were aligned against the human reference genome hg19 with BWA (12). Genomic variants were called with samtools and VarScan (13). Variant annotation was performed using SnpEff (14). Variant filtering was performed with Ingenuity® Variant Analysis™ (IVA) software (www.qiagen.com/Ingenuity, QIAGEN). Only variants with read depths of at least 10 and allele frequencies of at least 0.3 were evaluated. Common variants with allele population frequencies of more than 0.1% or homozygous counts of 5 or more in the ExAC or gnomAD databases were filtered out unless reported as disease-causing in the HGMD® (BIOBASE GmbH) or dbSNP databases (15, 16). Variants predicted to have low impact by at least 2 out of 3 scores calculated by SIFT, PolyPhen2, or CADD and present in population databases were also discarded (17–19). Remaining variants were manually evaluated in Integrative Genomics Viewer (http://www.broadinstitute.org/igv) to exclude variants in reads with low mapping quality (20). The analysis was then focused on variants in genes reported as causative for inborn errors of immunity in the last International Union of Immunological Societies (IUIS) guidelines, cancer-predisposition genes in children and in-house lists of genes possibly leading to immune dysregulation based on recent publications and close interactions with causative genes reported by IUIS (21, 22).

## Results

### Epidemiology and Clinical Manifestation

Our cohort of patients included 295 patients followed for 3,070 patient/years in total. The average ages at the first CVID-related symptoms and at the time of CVID diagnosis in a subgroup of CVID patients with malignancy were 34.2 and 38.3 years, respectively. A total of 25 malignancies were found in 22 patients (7.4% of all included patients) with SIR 6.3 (95% CI: 4.08–9.31). These cases included 6/25 (24.0%) gastric carcinoma (GC): SIR 5.7, 95% CI: 2.08–12.32, 4/25 (16.0%), B cell Non-Hodgkin lymphoma (B-NHL): SIR 5.5, 95% CI: 1.50–14.09, 5/25 (20.0%), Hodgkin lymphoma (HL):SIR 30.0, 95% CI: 9.73–69.93 and 10/24 (41.7%) other cancers (SIR 5.0, 95% CI: 2.40–9.16). These were two cases of spinocellular carcinoma, basocellular carcinoma, and T-cell lymphoma, and a single case each of tonsillar carcinoma, breast carcinoma, renal carcinoma and urine bladder cancer. Cancer duplicity was observed in 3 patients (gastric and tonsillar carcinoma, breast cancer and urothelial carcinoma, spinocellular, and gastric carcinoma). Malignancies were diagnosed in 16 males and 6 females; the average age at diagnosis was 52.3 years (15 years after the diagnosis of CVID and 19 years after the first symptoms). The average ages of manifestation of GC, B-NHL, and HL in CVID cancer group were 55–59, 35–39, and 40–44 years, respectively.

Autoimmune cytopaenias, including Immune thrombocytopenic purpura (ITP) and Autoimmune hemolytic anemia (AIHA), were the most common complications in CVID both with and without malignancy. They were found in 8/22 (36.4%) and 27/160 (16.9%), respectively. Interestingly, a strikingly increased risk of malignancy was detected in a subgroup of CVID patients with a history of ITP (RR 3.52, 95% CI 1.42–7.26). Cumulative risk (RR 4.53, 95% CI 1.23–11.59) observed for B-NHL and HL together was similar. However, no risk increase was observed for isolated HL, B-NHL, and GC, probably due to the small number of cancer events and because the direct association with other documented CVID-related comorbidities was not significant (data not shown).

As mentioned above, lymphomas and GC represent the most frequent malignancies diagnosed in CVID patients in our cohort. While five out of six patients with GC (4 males, 2 females) underwent surgery, one received palliative chemotherapy with FLOX regimen (fluorouracil, leucovorin, oxaliplatin) due to highly progressed disease at the time of diagnosis. Five out of six patients had deceased before the study initiation, 2/6 (33.3%) due to disease progression, 2/6 (33.3%) passed away after achieving more than 10 years survival, and one patient died because of malnutrition due to severe enteropathy 2 years after the diagnosis of lymphoma. All patients suffered from various gastrointestinal complications related to CVID, and the majority of them also had splenomegaly.

All 5 patients (4 males, 1 female) with HL received chemotherapy. The following chemotherapeutic regimens were used: R-CHOP (rituximab, cyclophosphamide, doxorubicin, vincristine, prednisone) in 2/5 (40%) of patients, BEACOPP (bleomycine, etoposide, doxorubicin, cyclophosphamide, vincristine, procarbazine, prednisone), eBEACOPP respectively, in 2/5 (40%) and DBVE-PC (adriamycin, bleomycine, vincristine, etoposide, prednisone, cyclophosphamide) in 1 patient (20%). No therapy-associated deaths or unexpected toxicities were noted. One patient died 3 years after treatment because of severe enteropathy, and 4/5 (80%) patients are still surviving today. All patients had previously described splenomegaly, and 3/5 (60%) had lymphadenopathy described prior to cancer diagnosis.

Similarly, all 4 patients (3 males, 1 female) with B-NHL received chemotherapy; no therapy-associated deaths or unexpected toxicities were noted. The following chemotherapeutic regimens were used: R-CHOP/CHOP [rituximab, cyclophosphamide, doxorubicin, vincristine, prednisone in 3/4 (75%) patients−2 with DLBCL (Diffuse large B-cell lymphoma) and in 1 patient with MALT (Mucosa-associated lymphoid tissue] lymphoma. A GMALL (German multicenter ALL) regimen was used in 1 patient with Burkitt lymphoma. Two of these patients had been regularly followed even prior to the diagnosis of B-NHL for lymphadenopathy and previously reported splenomegaly. All patients are still alive. T-NHL was diagnosed in 2 patients. The clinical features of CVID patients in whom lymphomas were diagnosed are summarized in Tables 2, 3. In this cohort, WES and detailed immunophenotyping were performed as part of further investigation (results presented further).

**Table 2:** Characteristics of the cohort of 11 CVID patients with lymphoma (M, Male; F, Female; LYM, Lymphadenopathy; SPLE, Splenomegaly; ITP, Idiopathic thrombocytopenic purpura; RTI, Respiratory tract infections; DBLCL lymphoma, Diffuse large B-cell lymphoma; MALT lymphoma, Mucosa-associated lymphoid tissue lymphoma; PTCL, Peripheral T-cell lymphoma; N/A, not applicable; Y, Yes; N, No; chemotherapeutic regimens are described in the results).

**Patient Nr**.	**Gender**	**Age at diagnosis of CVID**	**Manifestation**	**IgG serum level**	**Age at diagnosis of lymphoma**	**Type of lymphoma**	**Cause of death**	**Survival interval**	**Staging**	**Therapy**
1	M	35 years	RTI	1.9 g/l	57 years	T lymphoma	Infection	0 month	Died before staging	Died before treatment
2	M	53 years	LYM, SPLE	1.15 g/l	64 years	HL	Enteropathy	36 months	IVB	BEACOPP
3	F	41 years	ITP	3.89 g/l	58 years	HL	Alive	4 months	IIA	R-CHOP
4	M	18 years	RTI	2.43 g/l	45 years	DBLCL lymphoma	Alive	9 months	IIA	R-CHOP
5	M	39 years	LYM, SPLE	0.03 g/l	35 years	DBLCL lymphoma	Alive	96 months	IVB	R-CHOP
6	M	37 years	ITP	4.1 g/l	40 years	HL	Alive	12 months	IVA	R-CHOP
7	M	36 years	RTI	4.1 g/l	42 years	HL	Alive	6 months	IIIB	eBEACOPP
8	M	26 years	ITP	4.88 g/l	36 years	Burkitt lymphoma	Alive	25 months	IIIA	B-NHL GMALL
9	F	25 years	RTI	2.88 g/l	36 years	MALT lymphoma	Alive	145 months	IVA	R-CHOP
10	M	11 years	RTI	4.48 g/l	11 years	HL	Alive	204 months	IIIA	DBVE-PC
11	M	25 years	RTI	1.76 g/l	30 years	PTCL	Infection	9 months	IVA	CHOP

**Table 3:** Characteristics of CVID-related complications in a cohort of 11 patients with lymphoma (ITP, Immune thrombocytopenic purpura; AIHA, Autoimmune hemolytic anemia; RA, Rheumatoid arthritis; LIPS, Lymphocytic interstitial pneumonia; BE, Bronchiectasis; ACOS, Asthma-COPD overlap syndrome; EAA, Exogenous allergic alveolitis; NLH, Nodular lymphoid hyperplasia; IBD, Intestinal bowel disease; CG, Chronic gstritis; Y, Yes; N, No).

**Patient Nr**.	**Autoimmunity**	**Chronic lung disease**	**Enteropathy**	**Granulomatous complications**	**Lymph-adenopathy**	**Spleno-megaly**
1	AI thyreoiditis	LIPS	NLH	N	N	Y
2	N	BE	Celiac-like disease	N	Y	Y
3	ITP	BE	N	N	Y	Y
4	ITP, AIHA	LIPS	Celiac-like disease	N	Y	Y
5	N	N	N	N	Y	Y
6	ITP	N	N	N	N	Y
7	RA	N	N	N	N	Y
8	ITP, psoriasis	ACOS	N	N	N	Y
9	N	EAA	IBD-like disease	N	Y	N
10	N	BE, NLH	NLH, CG	N	Y	Y
11	N	BE	NLH	N	Y	N

### Immunophenotype

Parameters of cellular immunity were investigated, including T cell (CD4 T helpers as well as CD8 T cytotoxic cells), B cell and NK cell counts. No significant differences were registered between the absolute counts of T cells, T helper cells and T cytotoxic cells at the time of diagnosis of CVID in a cohort of patients with lymphoma compared to those without lymphoma. The T cellcounts were also well within the normal reference ranges (T cells 0.8−2.10E9/l, T helper cells 0.3−2.8E9/l, T cytotoxic cells 0.2−1.0E9/l). Unsurprisingly, the chemotherapeutic regiments for lymphoma led to skewing of T cell numbers (median 0.65E9/l, 95% CI 0.46–0.75 vs. 1.22E9/l, 95% CI 1.07–1.47, ^***^p = 0.0004), specifically T helper cells (median 0.34E9/l, 95% CI 0.14–0.36 vs. 0.56E9/l, 95% CI 0.53–0.76, ^***^p = 0.0004) and T cytotoxic cells (0.26E9/l, 95% CI 0.14–0.36 vs. 0.54E9/l, 95% CI 0.41–0.64, ^**^p = 0.006).

**Figure 1:**
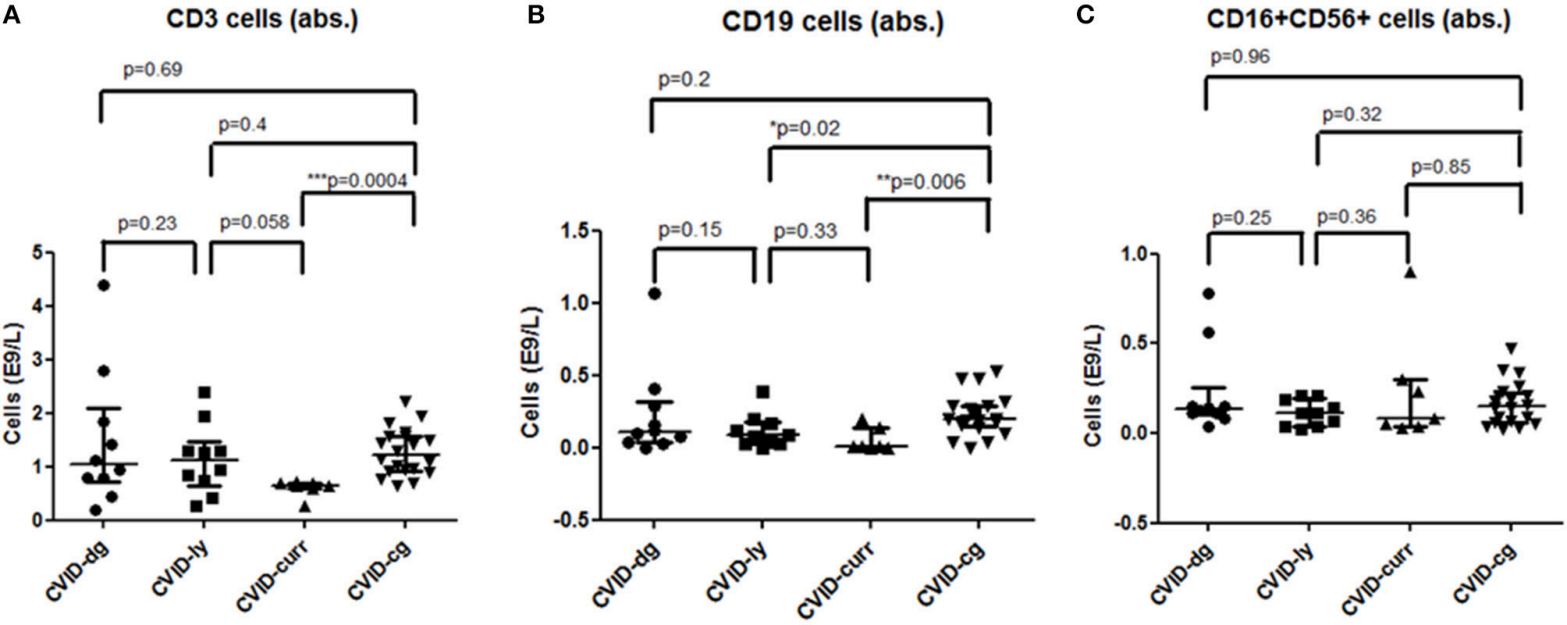
Absolute counts of **(A)** T (CD3+) cells, **(B)** B (CD19+) cells, and **(C)** NK (CD16+, CD56+) cells in a cohort of CVID patients with lymphoma at the time of diagnosis of CVID (CVID-dg), at the time of diagnosis of lymphoma (CVID-ly) and current values (CVID-curr) compared to the control group of CVID patients without lymphoma (CVID-cg);median and 95% CI are shown.

**Figure 2:**
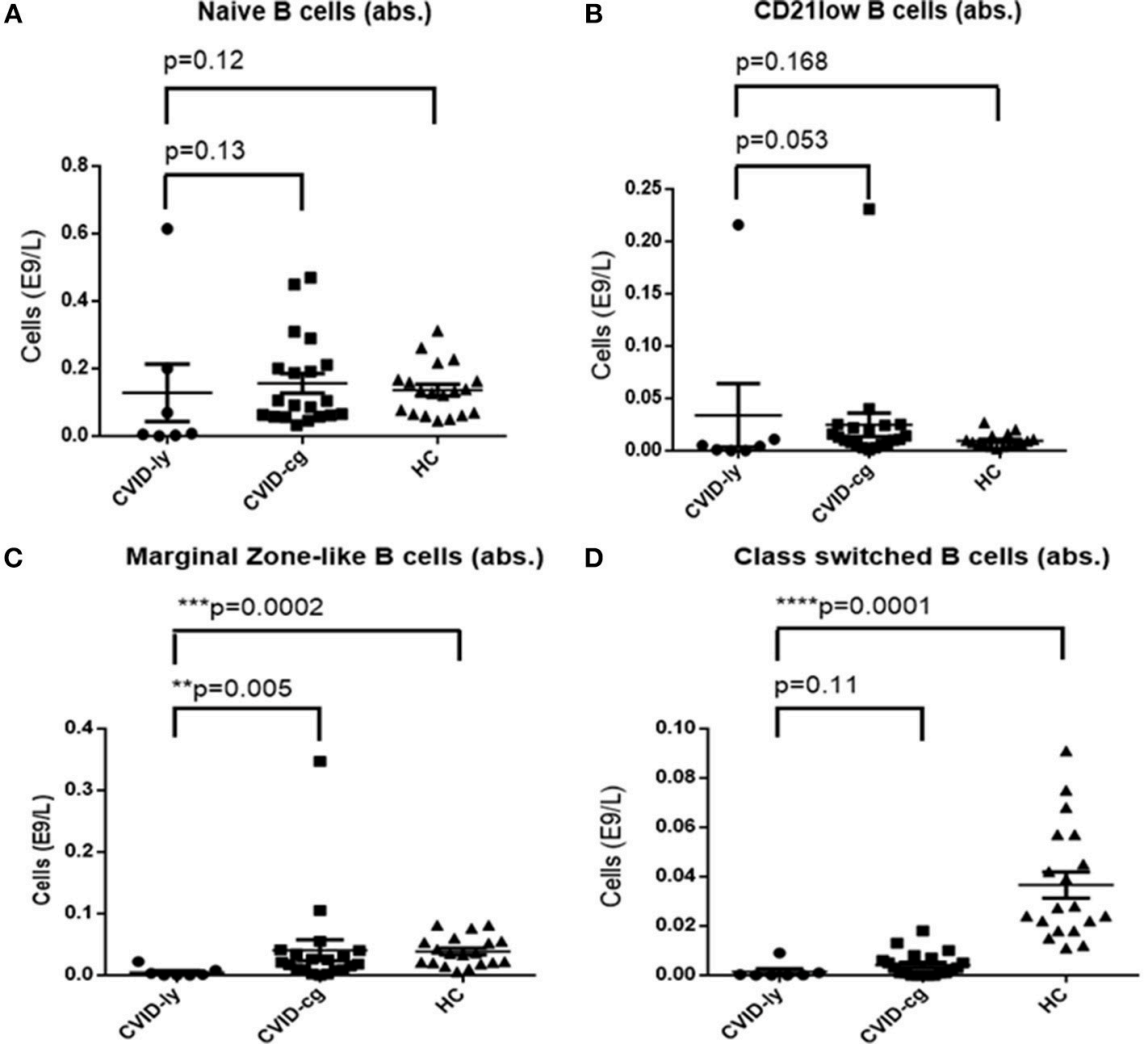
Absolute counts of **(A)** Naïve B cells, **(B)** CD21low B cells, **(C)** Marginal Zone-like B cells, and **(D)** Class-switched B cells in CVID patients with lymphoma upon chemotherapy (CVID-ly) compared to the compared to the control group of CVID patients without lymphoma (CVID-cg) and healthy controls (HC); median and 95% are shown.

The number of total B cells at the diagnosis of CVID did not differ significantly from the control group of CVID patients without lymphoma and from normal ranges. No significant difference was noted in the serum levels of IgG in the group of CVID patients with lymphoma (median 2.88 g/l, 95% CI 1.83–3.91, normal values 7.65–13.6 g/l) compared to the CVID control group (median 2.02 g/l, 95% CI1.63–3.24). In contrast, the number of total B cells at the diagnosis of lymphoma was reduced in the lymphoma group (median 0.01E9/l, 95% CI 0–0.13 vs. 0.195E9/l, 95% CI 0.16–0.29, ^**^p = 0.006). Absolute B cell counts were further depleted by the chemotherapy (median 0.11E9/l, 95% CI 0–0.46 vs. 0.08E9/l, 95% CI 0.03–0.197, ^*^p = 0.02). Post-therapeutic B cell lymphopenia (B cells count ≤ 0.03E9/l) was found in 6 patients. A complete total B cell count reconstitution was achieved in only 3 patients (median 0.33E9/l, range 0.137–0.654); however, mature forms of B cells, including marginal zone-like, class-switched cells and plasmablasts, remained reduced in these subjects (mean interval after chemotherapy 102 months, range 6–204). The remaining 3 patients failed to re-establish their B cell populations and continued to maintain severely reduced B cell compartments (mean 0.02E9/l, range 0.001–0.08; mean interval after chemotherapy 39 months, range 4–145). Concerning NK cells, their absolute counts were similar to CVID patients without lymphoma and the general population (normal range 0.05–1.0 E9/l) and remained unchanged throughout the disease course. Curiously, no NK cell depression was observed after the chemotherapy. The immunophenotype profiles are summarized in Figure 1, and the B cell subpopulations are shown in detail in Table 4 and Figure 2.

**Table 4:** B cell subpopulations in CVID patients with lymphoma post-chemotherapy (absolute counts in E9/L; reference values for general population in brackets; (↓), decreased count; (↑), increased count; N/A, value not available).

**Patient Nr**.	**CD21low (0.01–0.02)**	**Naïve (0.06–0.47)**	**Transitional (0.0–0.03)**	**MZ-like (0.01–0.08)**	**Class-switched (0.02–0.09)**
1	N/A	N/A	N/A	N/A	N/A
2	0.0252 (↑)	0.069	0.004	0.009 (↓)	0.002 (↓)
3	N/A	N/A	N/A	N/A	N/A
4	0.001 (↓)	0.005 (↓)	0.002	0 (↓)	0 (↓)
5	0.216 (↑)	0.614 (↑)	0.137 (↑)	0.022	0.001 (↓)
6	0 (↓)	0 (↓)	0	0 (↓)	0 (↓)
7	0.005 (↓)	0.2	0.045 (↑)	0.003 (↓)	0 (↓)
8	0.004 (↓)	0.0076 (↓)	0	0.0013 (↓)	0 (↓)
9	0.0002 (↓)	0.002 (↓)	0	0 (↓)	0 (↓)
10	0.011	0.069 (↓)	0.059 (↑)	0.008 (↓)	0.009 (↓)

### Whole Exome Sequencing

WES was performed in 10 out of 11 CVID patients with lymphoma in whom biological material for genetic testing was available. The WES results were divided into 5 groups. Gene variants previously described in association with CVID or in patients with inborn error of immunity (Group 1) were found in 4 patients. A novel heterozygous missense variant in *CTLA4* was identified inpatient Nr. 9, who developed B—NHL (MALT) at the age of 36 years. She was also followed and treated for lymphadenopathy and enteropathy (with features of IBD-like disease), which is in concordance with the expected phenotype of CTLA4 deficiency. The deleterious effect of the mutation was verified by determination of decreased basal and stimulated (CD3/CD28) expression of CTLA4 protein in the patient's T regulatory cells (CD4+CD127dimCD25+) (Supplementary Figure 1).

Another type of B—NHL (DBLCL) was diagnosed in patient Nr. 4 at the age of 45 years. A genetic variant in *PMS2* was found, which has an important role in the mismatch repair system and class switch recombination (23, 24). In addition to the lymphoma, the patient also manifested with a broad spectrum of non-infectious complications, including autoimmune cytopaenias (both AIHA as well as ITP), celiac–like disease and generalized lymphoproliferation, including lymphadenopathy, splenomegaly, and lymphocytic interstitial pneumonia.

The clinical manifestation of patient Nr. 10, who was found to harbor a *PIK3CD* mutation, corresponded with the previously described APDS (activated PI3K-delta syndrome) phenotype due to an activating mutation (2). He has been followed for generalized lymphadenopathy, splenomegaly, and nodular lymphoid hyperplasia (NLH) of the lungs and gastrointestinal tract since his childhood. This patient developed Hodgkin lymphoma at the age of 11 years.

Nodular lymphoid hyperplasia and lymphocytic interstitial pneumonia were also noted in patient Nr. 1, who was followed and treated for splenomegaly and autoimmune thyroiditis before the diagnosis of T cell lymphoma, which developed at the age of 57. A gene variant in *TNFRSF13B (TACI)*, known to increase susceptibility to CVID, was found. *TACI* variants are not regarded as disease-causing but rather as modifying (Group 2) (8).

Furthermore, several heterozygous variants were identified in genes associated with known primary immunodeficiencies that are inherited in an autosomal recessive manner, such as *LYST, LRBA, RAG1, EXTL3, and STX11* (Group 4). The clinical phenotype of these patients did not match the respective disease; nevertheless, we report these variants because of their rarity in the healthy population and their potentially damaging character predicted by *in silico* tools. Functional assays, which would elucidate the impact of these variants on protein function, were not performed, as they exceeded the scope of this study. The tumor DNA was not available for analysis of somatic “second-hit” mutations, which might explain the pathogenesis of some of the malignancies.

Variants in genes previously described in association with cancer susceptibility or as likely to increase the risk of cancer development, such as *BRCA1, RABEP1, EP300, KDM5A*, and others, were found in 6 out of 10 patients. They were divided into variants reported as pathogenic (Group 3) and variants of unknown significance and novel variants predicted as damaging *in-silico* (Group 5).The summary of WES results and a detailed description of the gene variants is presented in Table 5 and in Supplementary Table 1.

**Table 5:** Summary of whole exome sequencing results performed in CVID patients with lymphoma.

**Patient Nr**.	**Chromo-some**	**Gene symbol**	**Transcript variant**	**Protein variant**	**Geno- type**	**SIFT function prediction**	**SIFT score**	**Polyphen-2 function prediction**	**CADD score**	**ExAC Freq**.	**GnomAD Freq**.	**HGMD accession**
**GROUP 1: VARIANTS IN IUIS-CLASSIFIED GENES WITH LINKS TO CVID**												
4	7	*PMS2*	c.1687C>T	p.R563*	Het				34.000	0.002	0.001	CM102799
9	2	*CTLA4*	c.515C>G	p.S172W	Het	Damaging	0.00	Possibly Damaging	28.700			
10	1	*PIK3CD*	c.3061G>A	p.E1021K	Het	Tolerated	0.07	Probably Damaging	31.000		0.000	CM067447
**GROUP 2: MODIFIER GENES**												
1	17	TACI	c.310T>C	p.C104R	Het	Damaging	0.00	Probably Damaging	25.900	0.321	0.346	CM052924
**GROUP 3: CANCER SUSCEPTIBILITY GENES**												
3	17	*BRCA1*	c.547+14delG		Het				0.424	0.012	0.009	CD176513
6	17	*BRCA1*	c.5263_5264insC	p.Q652fs*74	Het				35.000	0.016	0.016	CI941841

## Discussion

Immune dysregulation associated with primary immunodeficiencies represents an increased risk of cancer development. We aimed to search for the occurrence of malignant diseases in a nationwide cohort of CVID patients, taking into account relevant epidemiology, immunophenotype, and the genetic background of the patients.

Similarly to published studies, we detected a higher incidence of malignancies among our CVID cohort (25–30). Also in alignment with previous reports, we noted a distinct spectrum of tumors in CVID patients, with Hodgkin and Non-Hodgkin lymphomas and gastric cancers being the most prevalent malignancies (Table 6 and Supplementary Table 2). The overall risk of malignancy was more than 6 times greater in comparison to the general population, while the specific risk of HL was as much as 30 times greater. Curiously, an over 3 times greater risk of malignancy was determined in a subgroup of CVID patients with a history of ITP. Moreover, we noted that the diagnosis of GC (average age 55–59 years vs. 70–74 in general population) and B-NHL (35–39 years vs. 65–69) was established at a much younger age compared to the Czech general population, while HL developed later in life compared to the healthy population (40–44 years vs. 30–34).

**Table 6:** Summary of tumor prevalence and SIR (Standardized Incidence Ratio, median and 95% Confidence Intervals (CIs) shown) in a cohort of 295 CVID patients.

**Tumor**	**Prevalence**	**SIR (95% CI)**
All tumors	25/295 (8.5%)	6.3 (4.08–9.31)
B cell lymphoma (all types)	9/295 (3.0%)	10.1 (4.61–19.12)
B cell non-Hodgkin lymphoma	4/295 (1.4%)	5.50 (1.50–14.09)
Hodgkin lymphoma	5/295 (1.7%)	30.0 (9.73–69.93)
Gastric cancer	6/295 (2.0%)	5.70 (2.08–12.32)
Other types of cancer	10/295 (3.4%)	5.0 (2.40–9.16)

Patients with CVID present with a characteristic immunophenotypic profile that is reflected in the diagnostic criteria of CVID. In this context, we specifically searched for potential differences between CVID patients with tumors and CVID patients who did not develop a malignant disease. Malignant hematologic diseases may, in general, reduce lymphocyte counts in up to 60% of patients, and lymphoma in particular may affect an entire spectrum of lymphocyte subpopulations, including CD4+, CD8+, CD19+, and CD56+ cells (31, 32). Nevertheless, in our cohort of CVID patients, we did not observe any significant differences between absolute or relative counts of CD3+, CD4+, CD8+, and CD56+ cells measured at the time of diagnosis of immunodeficiency and those measured at the time of diagnosis of lymphoma. Furthermore, the values of all T cell subpopulations and NK cells were similar to the control group of CVID patients without malignancy. In contrast, chemotherapy regimens had significant impacts on the CD4+ and CD8+ cell counts. A similar observation has already been published in patients with lymphomas without underlying primary immunodeficiencies who underwent chemotherapy with an R-CHOP protocol. In the study, a reduction of CD4+ absolute counts to values <0.343 × 10^9^/l was declared an independent negative prognostic factor with a significant impact on 5-year progression-free survival and overall survival (33). Indeed, in our CVID cohort with lymphoma, the median level of post-chemotherapy absolute numbers of CD4+ was very low, 0.343 × 10^9^/l, 95% CI 0.14–0.36 (R-CHOP being the regiment used in 5 out of 11 patient), which implies that a CVID population treated with chemotherapy should be prognostically regarded as a higher risk group. Quite unexpectedly, NK cell counts were not affected by the chemotherapy. However, the B cell compartment was profoundly depleted in all CVID patients who underwent chemotherapy. The total B cell count normalized in only 3/10 patients. However, even in those, selective reductions of mature forms (including class-switched, marginal zone-like B cells and plasmablasts) persisted. This observation was in striking contrast to patients with autoimmune diseases receiving anti-CD20 therapy (rituximab), in whom the reconstitution is achieved within 5–9 months in up to 90% of patients (34).

B cell deficiency seems to be the hallmark in CVID patients with lymphoma, as they presented with reduced B cell count even at the time of diagnosis of lymphoma, which decreased and remained persistently lower after chemotherapy. Despite this, neither B cell nor T cell detailed immunophenotyping provided a strong enough predictive tool for assessment of the cancerogenic predisposition of CVID patients. Therefore, we set out to search for possible genetic causes of malignancies in CVID using massive parallel sequencing. Out of 10 patients who were available for testing, we identified gene variants previously classified as associated with CVID in 4 patients, namely, *CTLR4, PIK3CD, PMS2*, and *TNFRSF13B*. It is noteworthy that mutations in *CTLA4* and *PIK3CD*, which account for the majority of currently known molecular causes of CVID (8), were both found among our small cohort of CVID patients.

*CTLA4* heterozygous mutation was first described as a cause of CVID-like syndrome that displayed a significant overlap with CVID phenotype, including hypogammaglobulinemia, low B cell counts and immune dysregulation with variable organ involvement (35). The clinical and laboratory spectra of *CTLA4* haploinsufficiency were described in detail in a recently published cohort of 133 patients. In this cohort, 8 mutation carriers developed lymphoma, and 3 had gastric cancer. Thus, our finding of a single*CTLA4* mutation among our small cohort corresponds well with this report. Furthermore, this particular patient also presented with IBD-like gastrointestinal disease that was retrospectively reclassified as a CTLA4-related gastrointestinal presentation. Interestingly, the spectrum of tumors found in the above mentioned CTLA4 study was limited to lymphomas and gastric carcinomas, which also correlates with our findings.

*PIK3CD* is a well-established genetic cause of APDS, activated PI3K-delta syndrome. Similarly toCLTA4, the clinical presentation of APDS overlaps significantly with the CVID phenotype, and a number of patients originally diagnosed with CVID were found to harbor mutations in *PIK3CD*. A large study including 53 patients with APDS reported lymphoma occurrence in 13% of patients (36). Furthermore, somatic mutations of *PIK3* were found in several types of HL and NHL, thus suggesting the role of PI3K signaling in tumorigenesis.

Finally, the *PMS2* protein is involved in complex mechanisms of DNA repair. As such, mutations in *PMS2* are directly associated with an increased risk of cancer (37). At the same time, mutations in *PMS2* were also implicated in class-switch recombination defects and impaired immunoglobulin production (36). Therefore, our finding of *PMS2* mutation among CVID patients with lymphoma corresponds well with these reports.

Overall, we suggest that each of these CVID-associated genes may also convey a predisposition to tumor development.

The role of the *TNFSF13B* molecule, also known as *TACI*, in CVID has long been discussed. *TACI* variants have been found to be associated with autoimmune complications in CVID (38). Moreover, given the involvement of *TACI* in B cell activation, its mutations might contribute to immune dysregulation and lymphoma development (39). However, *TACI* variants are regarded as modifier genes rather than a monogenic cause of CVID.

Apart of the above mentioned genetic findings, we identified several variants in genes involved in lymphogenesis and immune system regulation. Although sufficient data to postulate their role in immune deficiency or tumorigenesis are lacking, we report them along with our results for the sake of completeness, reflecting the previously described roles of heterozygous mutations in PID and possible epistatic roles of various genes in immune dysregulation (40–42).

Finally, we also detected several variants in genes involved in tumor surveillance in our cohort, such as BRCA1 and others listed in Supplementary Table 1. These variants were previously reported in patients with a broad spectrum of solid tumors (including breast, ovarian, colorectal cancer, and others) and may therefore represent another contributory mechanism of malignant susceptibility.

## Conclusion

Malignancies belong to the most severe non-infectious complications of common variable immunodeficiency disorder. The prevalence of malignancy in our CVID cohort was found to be more than 6 times greater than in the general population. The spectrum of cancers was characteristically narrow, involving mostly gastric cancers and lymphomas. Moreover, ITP was elucidated as a novel risk factor for malignancy in CVID patients. Post-treatment T and B cell lymphopaenias, associated with poorer prognosis, were found in a majority of CVID patients who received chemotherapy. Surprisingly, NK cells remained unaffected. WES analysis illustrated a wide heterogeneity of potential genetic background of the oncogenic predisposition among CVID patients and identified several causative or contributing gene variants, pointing toward immune system dysregulation. In the future, modern genetic analytic approaches applied on larger cohorts of CVID patients, along with the use of oncogenomic tools, will undoubtedly enable the identification of other CVID-associated genes with increased risk of cancer and elucidate their roles in tumorigenesis.

## Author Contributions

PK and TM conceived and designed the study, collected data, and drafted manuscript. JL, IM, DJ, JP, JV, AZ, MB, ZP, and AK collected and provided primary patient data. EF and MS performed analysis and interpretation of data from genetic testing (Whole exome sequencing). VK performed analysis and interpretation of immunophenotyping data. JH provided statistical analysis of the obtained data and its interpretation. TK and AS provided critical revisions of the manuscript and final approval of the version to be published.

### Conflict of Interest Statement

The authors declare that the research was conducted in the absence of any commercial or financial relationships that could be construed as a potential conflict of interest.
