# Supplementary material for: CVID-Associated Tumors: Czech Nationwide Study Focused on Epidemiology, Immunology, and Genetic Background in a Cohort of Patients With CVID
Source: Front Immunol. 2019 Jan 22;9:3135. doi: 10.3389/fimmu.2018.03135 (PMC6349737; doi:10.3389/fimmu.2018.03135)
Supplement: Supplementary file 1 [file Table_1.DOCX]

**Supplementary Table 1**

| **Group 4: Variants in IUIS-classified genes with different phenotypes** | | | | | |  | | | | | | | | | |
| --- | --- | --- | --- | --- | --- | --- | --- | --- | --- | --- | --- | --- | --- | --- | --- |
| **Patient Nr.** | **Chromo- some** | **Gene Symbol** | **Transcript Variant** | **Protein Variant** | **Geno- type** | | **SIFT Function Prediction** | | **SIFT Score** | **Polyphen-2 Function Prediction** | **CADD Score** | | **ExAC Freq.** | | **GnomAD Freq.** |
| **1** | 1 | *LYST* | c.7916T>A | p.L2639H | Het | |  | |  | Possibly Damaging | 24.400 | |  | |  |
| **2** | 8 | *EXTL3* | c.572C>T | p.T191I | Het | | Tolerated | | 0.19 | Possibly Damaging | 23.600 | |  | |  |
| **5** | 4 | *LRBA* | c.4048A>T | p.N1350Y | Het | | Damaging | | 0.00 | Probably Damaging | 31.000 | |  | |  |
|  | 6 | *STX11* | c.734T>C | p.I245T | Het | | Damaging | | 0.00 | Probably Damaging | 26.700 | |  | |  |
| **6** | 11 | *RAG1* | c.2774C>G | p.T925R | Het | |  | |  | Possibly Damaging | 22.900 | |  | |  |
| **Group 5: Variants in cancer susceptibility genes** | | | | | | | | | | | | | | | |
| **1** | 1 | *ABCA4* | c.2969G>T | p.G990V | Het | | Tolerated | 0.50 | | Possibly Damaging | 20.600 |  | |  | |
|  | 9 | *CNTRL* | c.2581G>A | p.A861T; p.A309T | Het | | Damaging | 0.00 | | Probably Damaging | 32.000 | 0.003 | | 0.002 | |
|  | 9 | *CNTRL* | c.3877G>A | p.V741M; p.V1293M | Het | | Damaging | 0.01 | | Possibly Damaging | 10.440 |  | |  | |
| **2** | 11 | *ARHGEF12* | c.1414G>T | p.V472F | Het | | Tolerated | 0.09 | | Probably Damaging | 27.700 |  | |  | |
| **3** | 12 | *KDM5A* | c.3934C>T | p.H1312Y | Het | | Damaging | 0.05 | | Probably Damaging | 23.700 |  | |  | |
|  | 17 | *RAD51D* | c.405+2T>C |  | Het | |  |  | |  | 24.600 |  | |  | |
| **4** | 7 | *PMS2* | c.1687C>T | p.R563* | Het | |  |  | |  | 34.000 | 0.002 | | 0.001 | |
| **5** | 6 | *STX11* | c.734T>C | p.I245T | Het | | Damaging | 0.00 | | Probably Damaging | 26.700 |  | |  | |
| **6** | 1 | *JUN* | c.-442C>T |  | Het | |  |  | |  | 20.300 |  | |  | |
|  | 16 | *SOCS1* | c.115delC | p.P39fs*46 | Het | |  |  | |  |  |  | |  | |
| **7** | 22 | *EP300* | c.2242-3C>A |  | Het | |  |  | |  | 17.800 |  | |  | |
| **9** | 15 | *KNL1* | c.453+3A>G |  | Het | |  |  | |  | 11.640 |  | |  | |
| **10** | 1 | *TNFRSF14* | c.88C>G | p.L30V | Het | | Damaging | 0.02 | | Probably Damaging | 21.500 |  | |  | |
|  | 7 | *HOXA13* | c.284C>A | p.A95E | Het | | Damaging | 0.01 | | Benign | 23.400 |  | | 0.004 | |

**Supplementary Table 1:** Supplementary results of Whole exome sequencing in CVID patients with lymphoma, including gene variants
