# Supplementary material for: CVID-Associated Tumors: Czech Nationwide Study Focused on Epidemiology, Immunology, and Genetic Background in a Cohort of Patients With CVID
Source: Front Immunol. 2019 Jan 22;9:3135. doi: 10.3389/fimmu.2018.03135 (PMC6349737; doi:10.3389/fimmu.2018.03135)
Supplement: Supplementary file 2 [file Table_2.DOCX]

| **Author** | **Population** | **Study**  **period** | **Number of patients** | **NHL** | **HL** | **GC** | **Other tumours** | **Total** |
| --- | --- | --- | --- | --- | --- | --- | --- | --- |
| Kinlen[24] | United Kingdom | 1957 -1981 | 220 | 3 (1.4%) | | 7 (3.1%) | 4  (1.8%) | 14 (6.4%) |
| Gathmann[23] | Europe | 2004 - 2012 | 902 | 23 (2.5%) | | 41 (4.5%) | | 64  (7.1) |
| Quinti[27] | Italy | 1999 - 2007 | 224 | 4 (1.8%) | 2 (0.9%) | 8 (3.6%) | 14 (6.25%) | 28 (12.5%) |
| Oksenhendler[26] | France | 2004 - 2007 | 252 | 16 (6.3%) | 0 | 26 (9.1%) | 42 (16.6%) | 84 (33.3%) |
| Resnick [28] | USA | 1973 - 2012 | 473 | 34 (7.1%) | 4 (0.8%) | 3 (0.6%) | 31 (6.3%) | 72 (15.2%) |
| Kokron[25] | Brasil | 1980 - 2003 | 71 | 3 (4.2%) | 1 (1.5%) | 2 (2.8%) | 6  (8.4%) | 12 (16.9%) |

**Supplementary Table 2A**

**Table 2A:** Epidemiology of CVID-associated tumours – the results of previously performed studies (NHL=Non-Hodgkin lymphoma, HL= Hodgkin lymphoma, GC= Gastric cancer)

**Supplementary Table 2B**

| **Author** | **Population** | **Study**  **period** | **Number of patients** | **NHL** | **HL** | **GC** | **Other tumours** | **Total** |
| --- | --- | --- | --- | --- | --- | --- | --- | --- |
| Kralickova, Milota | Czech Republic | 1997 - 2016 | 295 | 4  (1.4%) | 5  (1.7%) | 6  (2%) | 10  (3.4%) | 25  (8.5%) |

**Table 2B:** Epidemiology of CVID-associated tumours – the results of a Czech nationwide study (NHL=Non-Hodgkin lymphoma, HL= Hodgkin lymphoma, GC= Gastric cancer)
