# Supplementary figures and images for: CVID-Associated Tumors: Czech Nationwide Study Focused on Epidemiology, Immunology, and Genetic Background in a Cohort of Patients With CVID
Source: Front Immunol. 2019 Jan 22;9:3135. doi: 10.3389/fimmu.2018.03135 (PMC6349737; doi:10.3389/fimmu.2018.03135)

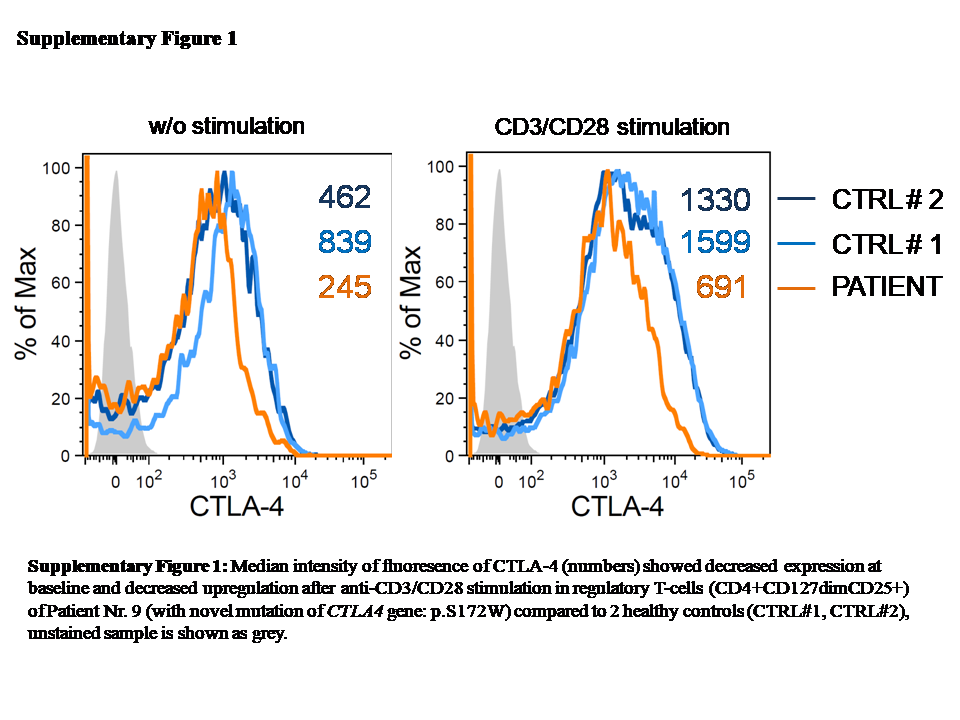

Supplement: Supplementary file 3 [file Image_1.tif]
